# SARS-CoV-2 Employ BSG/CD147 and ACE2 Receptors to Directly Infect Human Induced Pluripotent Stem Cell-Derived Kidney Podocytes

**DOI:** 10.3389/fcell.2022.855340

**Published:** 2022-04-20

**Authors:** Titilola D. Kalejaiye, Rohan Bhattacharya, Morgan A. Burt, Tatianna Travieso, Arinze E. Okafor, Xingrui Mou, Maria Blasi, Samira Musah

**Affiliations:** ^1^ Department of Biomedical Engineering, Pratt School of Engineering, Duke University, Durham, NC, United States; ^2^ Center for Biomolecular and Tissue Engineering, Duke University, Durham, NC, United States; ^3^ Division of Infectious Diseases, Department of Medicine, Duke University School of Medicine, Durham, NC, United States; ^4^ Duke Human Vaccine Institute, Duke University Medical Center, Durham, NC, United States; ^5^ Division of Nephrology, Department of Medicine, Duke University School of Medicine, Durham, NC, United States; ^6^ Developmental and Stem Cell Biology Program, Duke University, Durham, NC, United States; ^7^ Department of Cell Biology, Duke University, Durham, NC, United States

**Keywords:** SARS-CoV-2, podocytes, S-pseudotyped virus, ACE2, BSG/CD147, organotropism, kidney disease, *in vitro* disease model

## Abstract

Severe acute respiratory syndrome coronavirus 2 (SARS-CoV-2) causes the Coronavirus disease 2019 (COVID-19), which has resulted in over 5.9 million deaths worldwide. While cells in the respiratory system are the initial target of SARS-CoV-2, there is mounting evidence that COVID-19 is a multi-organ disease. Still, the direct affinity of SARS-CoV-2 for cells in other organs such as the kidneys, which are often targeted in severe COVID-19, remains poorly understood. We employed a human induced pluripotent stem (iPS) cell-derived model to investigate the affinity of SARS-CoV-2 for kidney glomerular podocytes, and examined the expression of host factors for binding and processing of the virus. We studied cellular uptake of the live SARS-CoV-2 virus as well as a pseudotyped virus. Infection of podocytes with live SARS-CoV-2 or spike-pseudotyped lentiviral particles revealed cellular uptake even at low multiplicity of infection (MOI) of 0.01. We found that direct infection of human iPS cell-derived podocytes by SARS-CoV-2 virus can cause cell death and podocyte foot process retraction, a hallmark of podocytopathies and progressive glomerular diseases including collapsing glomerulopathy observed in patients with severe COVID-19 disease. We identified BSG/CD147 and ACE2 receptors as key mediators of spike binding activity in human iPS cell-derived podocytes. These results show that SARS-CoV-2 can infect kidney glomerular podocytes *in vitro* via multiple binding interactions and partners, which may underlie the high affinity of SARS-CoV-2 for kidney tissues. This stem cell-derived model is potentially useful for kidney-specific antiviral drug screening and mechanistic studies of COVID-19 organotropism.

## Introduction

The Coronavirus disease 2019 (COVID-19)—caused by the severe acute respiratory syndrome coronavirus 2 (SARS-CoV-2)—has affected more than 441 million people and caused over 5.9 million deaths worldwide (Johns Hopkins University and Medicine, 2021) (retrieved 3 March 2022). Although SARS-CoV-2 primarily infects cells in the respiratory tract, other tissues and organs have also been shown to be highly vulnerable to the virus resulting in a broad array of complications in the renal, cardiovascular, gastrointestinal and nervous systems (Gupta et al., 2020; Naicker et al., 2020; Zhang et al., 2020) particularly in elderly cases and in those with comorbidities (Deshmukh et al., 2021). Several *in vitro* studies have examined the impact of SARS-CoV-2 infection in lung and cardiac cells (Sharma et al., 2020; Sungnak et al., 2020; Yang et al., 2020; Marchiano et al., 2021). SARS-CoV-2 viral RNA from tissues of living and dead patients of COVID-19 has been detected in multiple organs including the kidneys (Puelles et al., 2020; Musah, 2021; Peiris et al., 2021). Intriguingly, acute kidney injury (Akilesh et al., 2021) and cardiac injury are common in COVID-19 patients (Cheng et al., 2020; Singh et al., 2020; Diao et al., 2021; Sharma et al., 2021) and have been associated with increased morbidity and mortality (Braun et al., 2020; Shi et al., 2020; Peiris et al., 2021). Additionally, collapsing glomerulopathy or COVID-19-associated nephropathy (COVAN) has been reported in COVID-19 patients (Velez et al., 2020; Sharma et al., 2021). COVAN resembles human immunodeficiency virus (HIV) -associated nephropathy (HIVAN), a kidney disease caused by HIV infection (Wyatt et al., 2008) resulting in CKD and kidney failure (Genovese et al., 2010). Notably, collapsing glomerulopathy constitutes a new renal manifestation of COVID-19 (Akilesh et al., 2021) that may also arise from genetic predisposition (Wu et al., 2020; Sharma et al., 2021).

Within the kidney, podocytes and proximal tubules play important roles in renal filtration, reabsorption and excretion (Pan et al., 2020). The glomerulus, a network of capillaries, is the primary site for blood filtration. The glomerular filtration barrier consists of interdigitated podocytes separated from fenestrated glomerular endothelial cells by the glomerular basement membrane (GBM), all of which function together to facilitate the selective filtration of toxins and waste from the blood (Lennon et al., 2014; Musah et al., 2017; Petrosyan et al., 2019). The kidney’s glomerular podocytes are particularly vulnerable to bacterial and viral attacks and injury which can result in retraction of podocyte foot processes and effacement, causing abnormal leakage of proteins into the urine (proteinuria) (Jefferson et al., 2011). Given that SARS-CoV-2 has been found in nephrin-positive cells of the kidneys of COVID-19 patients (Puelles et al., 2020), we hypothesized that podocytes could be direct targets for SARS-CoV-2 infection.

SARS-CoV-2 organotropism (cell types or tissues permissive to viral infection) is influenced by the expression of suitable receptors on the cell surface and the presence of a host-encoded protease proximally positioned to the site of receptor binding to enable cleavage of the Spike (S) protein for viral processing (Singh et al., 2020; Tang et al., 2020). Angiotensin-Converting Enzyme 2 (ACE2) is widely recognized as a key receptor for SARS-CoV-2 binding to cells and tissues (Fehr and Perlman, 2015; Hoffmann et al., 2020; Lan et al., 2020; Moore and June, 2020; Walls et al., 2020). Basigin (BSG, also known as CD147 or EMMPRIN), a transmembrane glycoprotein has also been shown to be an alternate route for SARS-CoV and SARS-CoV-2 invasion of host cells (Chen et al., 2005; Wang et al., 2020). For instance, BSG/CD147 is shown to interact with S protein *in vitro* and facilitate entry of SARS-CoV and SARS-CoV-2 in Vero and HEK 293T cell (Vankadari and Wilce, 2020; Wang et al., 2020). Other receptors such as Neuropilin 1, a pleiotropic transmembrane peptide growth factor in fibroblasts, endothelial cells, and hepatocytes (Glinka et al., 2010) has been shown to enhance SARS-CoV-2 entry and infectivity when co-expressed with ACE2 and TMPRSS2 (Cantuti-Castelvetri et al., 2020). Additionally, CD209/DC-SIGN interacts with spike receptor binding domain (S-RBD) and can mediate SARS-CoV-2 entry into human cells (Amraei et al., 2021). Once the viral spike protein binds to the host receptor (Wrapp et al., 2020), the activity of proteases such as Transmembrane Serine Protease 2 (TMPRSS2) or Cathepsin L (CTSL) promote fusion and internalization of the receptor-viral spike complex (Wysocki et al., 2021).

Understanding the susceptibility of organ-specific cell types to SARS-CoV-2 infection and COVID-19 disease mechanisms rely on the availability of robust experimental models that can closely mimic the functional phenotype and developmental status of human cells and tissues. However, functional *in vitro* models are lacking for many tissues. For instance, the lack of appropriate *in vitro* models contributes to the poor understanding of how SARS-CoV-2 invades the human kidney tissues including the specialized group of visceral epithelial cells called podocytes. Podocytes encase the glomerular capillaries and play a vital role in regulating the removal of toxins and waste products from blood. These cells, are also susceptible to disease including those arising from drug toxicities and viral infections. As a result, there is a dire need to study SARS-CoV-2 infection in human kidney podocytes. Because stem cells can self-renew indefinitely and differentiate into almost any cell type when provided appropriate signals, they serve as virtually unlimited supply of organ-specific cells including podocytes (Musah et al., 2017). Derivatives of human pluripotent stem cells have also been used for disease modelling and drug discovery assays (Ilic and Ogilvie, 2017; Bhattacharya et al., 2021; Okafor et al., 2021; Kalejaiye et al., 2022). We previously developed a method to directly differentiate human iPS cells into cells that exhibit morphological, molecular, and functional characteristics of the mature human kidney glomerular podocytes (Musah et al., 2017; Musah et al., 2018; Burt et al., 2020). Herein, we employed this model to study the susceptibility of human kidney podocytes to SARS-CoV-2 infection. We also investigated the expression and involvement of host receptors and processing enzymes in SARS-CoV-2 binding to human kidney podocytes.

## Materials and Methods

### Cell Culture

All cell lines used for this study were obtained under appropriate material transfer agreements and approved by all involved institutional review boards. All cells were tested for and shown to be devoid of *mycoplasma* contamination (*Mycoplasma* PCR Detection Kit from abm, G238). Human colon epithelial (Caco-2) (ATCC, HTB-37) and human embryonic kidney (HEK 293T) (ATCC, CRL-3216) cell lines were cultured in high-glucose Dulbecco’s Modified Eagle Medium (DMEM; Gibco, 12634010) media supplemented with 10% fetal bovine serum (FBS; Gibco; 10082147) with L-Glutamine (Gibco; 25030081) and 1X Penicillin/Streptomycin (Gibco; 15140122). HEK 293T cells were split (1:10) while Caco-2 cells were split (1:5) every 3 days. Human lung (Calu-3) (ATCC, HTB-55) cells were cultured in Minimum Essential Media (MEM) (Gibco; 11095080) supplemented with 10% FBS with 1 mM sodium pyruvate (Gibco; 11360070), MEM non-essential amino acids (NEAA) (Gibco; 11140050) with 1X Penicillin/Streptomycin. Human induced pluripotent stem (Human iPS) cell line used for this study (PGP1—the Personal Genome Project (Ball et al., 2012)) were tested and shown to be free of *mycoplasma* contamination. The cell line had normal karyotype. Human iPS cells were cultured in mTeSR1 (StemCell Technologies; 85870) medium without antibiotics and split (1:6) every 4–5 days. All cells were incubated in a 37°C incubator with 5% CO_2_.

### Differentiation of Human iPS Cells Into Podocytes

Mature human glomerular podocytes were generated using previously published protocol (Musah et al., 2017; Musah et al., 2018; Burt et al., 2020). Briefly, human induced pluripotent stem (iPS) cells cultured on Matrigel-coated plates were dissociated with warm enzyme-free dissociation buffer (Gibco; 13150-016) and centrifuged twice at 200x*g* for 5 min each in advanced DMEM/F12 (Gibco; 12634010). The DMEM/F12 was aspirated off and the cells were resuspended in mesoderm induction media (consisting of DMEM/F12 with GlutaMax (Gibco; 10565042) supplemented with 100 ng/ml activin A (Invitrogen; PHC9564), 3 μM CHIR99021 (Stemgent; 04-0004), 10 μM Y27632 (TOCRIS; 1254) and 1X B27 serum-free supplement (Gibco; 17504044) and plated at a seeding density of 100,000 cells per well of a 12-well plate. The cells were cultured in the mesoderm induction medium for 2 days with daily medium change and after 2 days, intermediate mesoderm differentiation was initiated by feeding the cells with intermediate mesoderm induction medium (containing DMEM/F12 with GlutaMax supplemented with 100 ng/ml BMP7 (Invitrogen; Phc9543), 3 μM CHIR99021 and 1X B27 serum-free supplement) for a minimum of 14 days. Podocyte induction was initiated by dissociating the intermediate mesoderm cells with 0.05% trysin-EDTA (Gibco; 25300-054) for 5 min with subsequent quenching of the enzyme with 10% FBS in DMEM/F12 (trypsin neutralizing solution). Adhered cells were gently scraped using a cell lifter and the cell suspension was pipetted up and down using a P1000 barrier tip to dislodge cells and obtain individualized cells. The cell suspension was transferred into a 50 ml falcon tube containing 30 ml DMEM/F12 and centrifuged twice at 200x*g* for 5 min each. The cell pellet was resuspended in podocyte induction mediaand plated on a freshly prepared laminin-511-E8-coated plates at a seeding density of 100,000 cells per well of a 12 well plate. The cells were fed podocyte induction media containing advanced DMEM/F12 with GlutaMax supplemented with 100 ng/ml BMP7, 100 ng/ml activin A, 50 ng/ml VEGF (Gibco; PHC9394), 3 μM CHIR99021, 1X B27 serum-free supplement, and 0.1 μM all-trans retinoic acid (Stem Cell Technologies; 72262) for 5 days. Mature podocytes were maintained in CultureBoost-R (Cell Systems; 4Z0-500).

### Production of SARS-CoV-2 S-Pseudotyped Lentiviral Particles

The SARS-CoV-2 S-pseudotyped lentiviral particles were generated by transfecting HEK 293T cells. Briefly, HEK 293T cells were seeded in DMEM-10 growth media in 75 cm^3^ flask and propagated to about 65%–75% confluent. The cells were then transfected with the plasmids required for the lentiviral production using Lipofectamine 3000 reagent (Invitrogen; L3000015) in Opti-MEM (Gibco; 31985070) following manufacturer’s instructions. Briefly, 20 µg of plasmid DNA (total plasmid mix) per flask was mixed in Opti-MEM and P3000 reagent and incubated at room temperature for 5 min. Plasmids mix for each transfection consisted of psPax2 (packaging; Addgene plasmid # 12260), pCMV-SCoV-2S (Spike envelope plasmid; Sinobiologicals—# VG40589-UT) and pLJM1-EGFP (reporter; Addgene plasmid #19319) in a ratio of 1:1:2, respectively. After 5 min incubation, the plasmid DNA mix in Opti-MEM- P3000 media was then mixed with the transfection reagent (Opti-MEM and Lipofectamine 3000 reagent) and incubated at room temperature for 10-15 min. Appropriate volumes of transfection mixture was used to transfect HEK 293Tcells in each flask and incubated in a 37°C incubator with 5% CO_2_ for 6 h. Lentivirus pseudotyped with the vesicular stomatitis virus spike G (pCMV-VSV-G; Addgene plasmid # 8454) was used as positive control and a “bald” lentivirus lacking the envelope was used as a negative control.

At 6 h post-transfection, the culture medium was replaced with fresh pre-warmed DMEM-10. After an additional 24 h (30 h post-transfection) and 48 h (54 h post-transfection), the lentiviral particles were harvested by collecting the supernatant from each flask, centrifuged at 1000x*g* for 5 min and filtered through a 0.45 µm SFCA low protein-binding filter. Samples were then subjected to ultracentrifugation over a 28% sucrose cushion (Sucrose/PBS; Sigma S7903-1KG) at 100,000x*g* for 3 h at 4°C. The pellet was resuspended in 1X Tris buffered saline (TBS, Bio-Rad; 1706345), and then aliquoted and stored at −80°C to avoid repeated freeze-thaw cycles.

### Infection of Cells With S-Pseudotyped Virus

Cells were cultured in the appropriate culture media and infected with S-pseudotyped, positive control or bald virus in the presence of polybrene (Sigma; TR-1003) to a final concentration of 5 µg/ml.

### SARS-CoV-2 Expansion in Vero E6 Cells and Titration

All experiments with the live virus were performed under Biosafety Level 3 (BSL-3) in the Duke Regional Biocontainment Laboratory at the Duke Human Vaccine Institute (DHVI) in compliance with the BSL-3 laboratory safety protocols and guidelines from the CDC for handling SARS-CoV-2.

SARS-CoV-2 USA-WA1/2020 (BEI Resources; NR-52281) was propagated in Vero E6 cells at a MOI of 0.001 in DMEM supplemented with 2% FBS, 1X Penicillin/Streptomycin, 1 mM Sodium pyruvate and 1X Non-Essential Amino Acid (NEAA) at 37°C in 5% CO_2_. Four days post infection (pi), supernatant containing the released virus were harvested, centrifuged at 1,500 rpm for 5 min and filtered through a 0.22 µM filter. Samples were aliquoted and stored at −80°C until further use.

Plaque assay was done to determine the titer of the viral stock. Briefly, 0.72 × 10^6^ Vero E6 cells were seeded in 24 well plates. The virus stock was diluted serially (10-fold), and the dilutions were used to infect monolayer of Vero E6 cells at 37°C in 5% CO_2_. After an hour of incubation, cells were overlayed with media containing carboxy-methyl cellulose (CMC) (0.6% CMC), MEM supplemented with 2% fetal bovine serum (FBS), 1 mM sodium pyruvate (Gibco), 1X NEAA (Gibco), 0.3% sodium bicarbonate (Gibco) and 1X GlutaMAX (Gibco) with 1X Penicillin/Streptomycin. After 4 days of incubation at 37°C in 5% CO_2_, cells were stained with 1% crystal violet in 10% neutral buffered formalin (NBF) and the number of plaque forming units per ml (pfu/ml) was determined.

### Infection of Human iPS Cell-Derived Podocytes With Live Virus

1 × 10^5^ intermediate mesoderm cells were differentiated to podocytes (per well of a 12 well plate). After 5 days of induction, podocytes were incubated with the SARS-CoV-2 virus at an MOI 0.01, 0.1 or 1.0 at 37°C and 5% CO_2_ with intermittent plate rocking. To obtain the desired MOI, SARS-CoV-2 was diluted in CultureBoost-R and incubated with the podocytes for 1 h at 37°C. After 1 h of incubation, the virus-containing supernatant was aspirated, and cells were washed twice with 1X PBS. Fresh maintenance medium was then added, and cells incubated for either 24, 48 or 72 h at 37°C and 5% CO_2_. Uninfected controls were incubated with CultureBoost-R only.

### Infectious Viral Titer of Supernatant

Cellular supernatant was collected from podocytes infected with SARS-CoV-2 virus at MOI of 0.01, 0.1 and 1.0, at 24 hrs, 48 and 72 hrs post infection. The supernatant was clarified by centrifugation at 1,500 rpm for 5 min and the infectious viral titer was measured by plaque assay as described above.

### qRT-PCR for Detection of Intracellular and Cell-free Viral RNA

SARS-CoV-2 RNA was extracted from the supernatant or cell pellet of infected podocytes using the QIAamp viral RNA mini kit (Qiagen; 52904). qRT-PCR was performed with primers specific for target genes (see Supplementary Table S1 for the list of primers) using the Luna universal One-Step RT-qPCR kit (NEB; E3005). Experiment was performed using the QuantStudio3 (Applied Biosystems) with the following thermal cycling steps; 55°C for 10 min, 95°C for 1 min and 40 cycles of 95°C for 10 s and 60°C for 1 min according to manufacturer’s protocol.

### qRT-PCR and qPCR Analysis of Infected Cells

Cell pellets were washed and lysed using RA1 RNA extraction buffer and purified using the NucleoSpin RNA kit (MAcherey-Nagel; 740955.250) following the manufacturer’s instructions. RNA from infected and control podocytes were harvested using NucleoSpin RNA kit. The RNA was quantified by nanodrop (Thermo Fisher). 0.5–1 µg of RNA was converted to cDNA for qPCR. cDNA synthesis was done using SuperScript III Reverse Transcriptase (Invitrogen; 18080-085) and qPCR was performed using qPCR SYBR Master Mix (Promega; A6001). Quantitative PCR was performed with QuantStudio3 (Applied Biosystems) using the thermal cycling steps; 50°C for 2 min, 95°C for 10 min and 40 cycles of 95°C for 15 s and 60°C for 1 min. Delta cycle threshold (ΔCt) was determined relative to GAPDH. Viral RNA from pseudovirus infected cells was also quantified by qRT-PCR using the Lenti-X qRT-PCR titration kit (Clontech; 631235) following manufacturer’s instruction. Primer sequences are provided in the Supplementary Table S1.

### Western Blot Analyses

For Western blotting, cells were first lysed using RIPA buffer (Sigma; R0278-500ML) supplemented with protease inhibitor cocktail (Roche) at 4°C with shaking for 30 min for protein extraction. Pierce BCA protein assay Kit (Thermo Fisher; 23227) was used for protein quantification. 15 µg of the extracted protein samples were boiled for 5 min at 95°C in 1X Laemlli buffer (BioRad; 1610747), run on mini-PROTEAN TGX precast gels (Bio-Rad; 4568083) and then transferred to PVDF membrane blot (Bio-Rad; 1620175). The blots were blocked in 5% non-fat milk made in TBS-T (50 mM Tris-HCl, 150 mM NaCl, 0.1% Tween-20) for 1 h and incubated with the primary antibodies in blocking buffer overnight at 4°C (see Supplementary Table S2 for the antibody dilutions). The next day, horseradish-peroxidase-conjugated rabbit anti-goat (R&D Systems; HAF017), goat anti-rabbit (CST; 7074) or goat anti-mouse (CST; 7076) antibody was added, and the blot was incubated for 1 h at room temperature. The membranes were developed with the SuperSignal West Femto substrate (Thermo Fisher; 34095) by following manufacturer’s protocol. The chemi-luminiscent signals were acquired using a GelDoc Imager (Bio-Rad).

### Identification of Spike Associated Host Factors Expressed by Podocyte

To identify podocyte host factors that could facilitate viral entry and replication, we integrated the BioGRID interaction database with transcriptomic data previously generated in our lab. BioGRID is an expansive database of experimentally verified protein-protein and genetic interactions as assembled and curated from tens of thousands of studies (Oughtred et al., 2019). Firstly the latest release of the BioGRID interaction database (as at when this study was carried out) for coronaviruses was downloaded from the archive at https://downloads.thebiogrid.org/Download/BioGRID/Release-Archive/BIOGRID-3.5.188/BIOGRID-CORONAVIRUS-3.5.188.tab3.zip. The interaction network file was then opened using Cytoscape (v3.8.0) and filtered to obtain only edges linking human proteins to the SARS-CoV2 spike protein.

We extracted podocyte gene expression data for spike-binding proteins and integrated it with the network table obtained from BioGRID. The microarray transcriptomic data for human iPS cell-derived podocytes used in this study had been generated in a previous study (Musah et al., 2018). The podocyte microarray gene expression data were analyzed using standard pipeline. Briefly, the raw expression data were normalized by robust multiarray averaging (Irizarry et al., 2003) and the Human Gene 2.0 ST Affymetrix array mapping obtained from the ENSEMBL mart database was used to map probe IDs to gene IDs. The podocyte transcriptomic data was analyzed using the Bioconductor packages, oligo (v3.11), biomaRt, and pd.hugene.2.0.st (Carvalho and Irizarry, 2010). The expression data for these proteins were then used to annotate a network visualization of these interactions on Cytoscape.

### Immunofluorescence Imaging

For immunofluorescent imaging, human iPS cell-derived podocytes (infected and control) were fixed with 4% paraformaldehyde (PFA) in PBS for 20–30 min at room temperature and permeabilized using 0.125% Triton X-100 (Sigma-Aldrich) in PBS for 5 min. Cells were blocked with 1% BSA/PBS-T for 30 min at room temperature and then incubated with primary antibody diluted in the blocking buffer overnight at 4°C. After overnight incubation, cells were incubated with Alexa Fluor-488 or Alexa Fluor-594 donkey (Invitrogen, 1:1000) secondary antibodies diluted in blocking buffer for 1 h at room temperature. Cells were afterwards counterstained with 4′,6-diamidino-2-phenylindole (DAPI, Invitrogen, D1306). The primary antibodies used were anti-Nephrin (Progen, GP-N2); anti-Podocin (Abcam, ab50339); anti-SARS-CoV2 spike (ProSci, 3525); anti-SARS-CoV-2 N protein (Sinobiological, 40143-R019); anti-GFP (Millipore, SAB4301138); Human/Mouse/Rat/Hamster ACE-2 (R&D systems, AF933), Human TRA-1-85/CD147 (R&D systems, MAB3195), Cathepsin L (Santa Cruz Biotechnology, sc-32320), TMPRSS2 (Santa Cruz Biotechnology, sc-515727) and DC-SIGN/CD209 (Santa Cruz Biotechnology, sc-65740). Images were acquired using an M7000 epifluorescence microscope (Invitrogen, AMF7000) equipped with 10×/0.30 LWDPH with 7.13 mm WD and 20×/0.45 LWDPH with 6.12 mm WD objectives. Confocal images were captured using a Zeiss 880 inverted confocal Airyscan with a 10×/0.30 EC Plan-Neofluar air lens with 5.2 mm objective at the Duke Light Microscopy Core Facility.

### Blocking of ACE2 and BSG/CD147 Protein With Antibodies

For the blocking of ACE2 and/or BSG/CD147 epitope, we infected podocytes with the S-pseudotyped lentivirus. Approximately 1.5 h prior to infecting cells, antibody dilutions were prepared in the CultureBoost-R. We performed the blocking experiment using an ACE2 polyclonal goat antibody (R&D systems; AF933) and CD147 (BSG) mouse monoclonal antibody (Human TRA-1-85/CD147 MAb (Clone TRA-1-85)- R&D systems; MAB3159). The human iPS cell-induced podocytes were pre-treated with serial dilutions of ACE2 antibody, BSG/CD147 antibody or both for 1 h. Unblocked cells and uninfected (mock) cells were used as control. After 1 h of incubation, the pseudoviral particles (MOI- 0.02) were added to each well and incubated for 48–60 h. After 60 h, cells were washed and lysed in RNA extraction buffer. RNA was purified using the Macherey Nagel RNA extraction kit following manufacturer’s instruction and viral RNA uptake was quantified using the Luna universal One-Step RT-qPCR kit (NEB; E3005).

### Quantification and Statistical Analysis

All experiments were done in 3 independent biological replicates unless otherwise indicated. N = 3. One-way analysis of variance (ANOVA) with Šidák’s posttest or multiple *t*-test was used to test for statistical significance. Only *p* values of 0.05 or lower were considered statistically significant (*p* > 0.05 [ns, not significant], *p* < 0.05 [*], *p* < 0.01 [**], *p* < 0.001 [***], *p* < 0.0001 [****]). For all statistical analyses, the GraphPad Prism 9 software package was used.

## Results

### Human iPS Cell-Derived Podocytes Are Permissive to S-Pseudotyped Viral Infection

**FIGURE 1:**
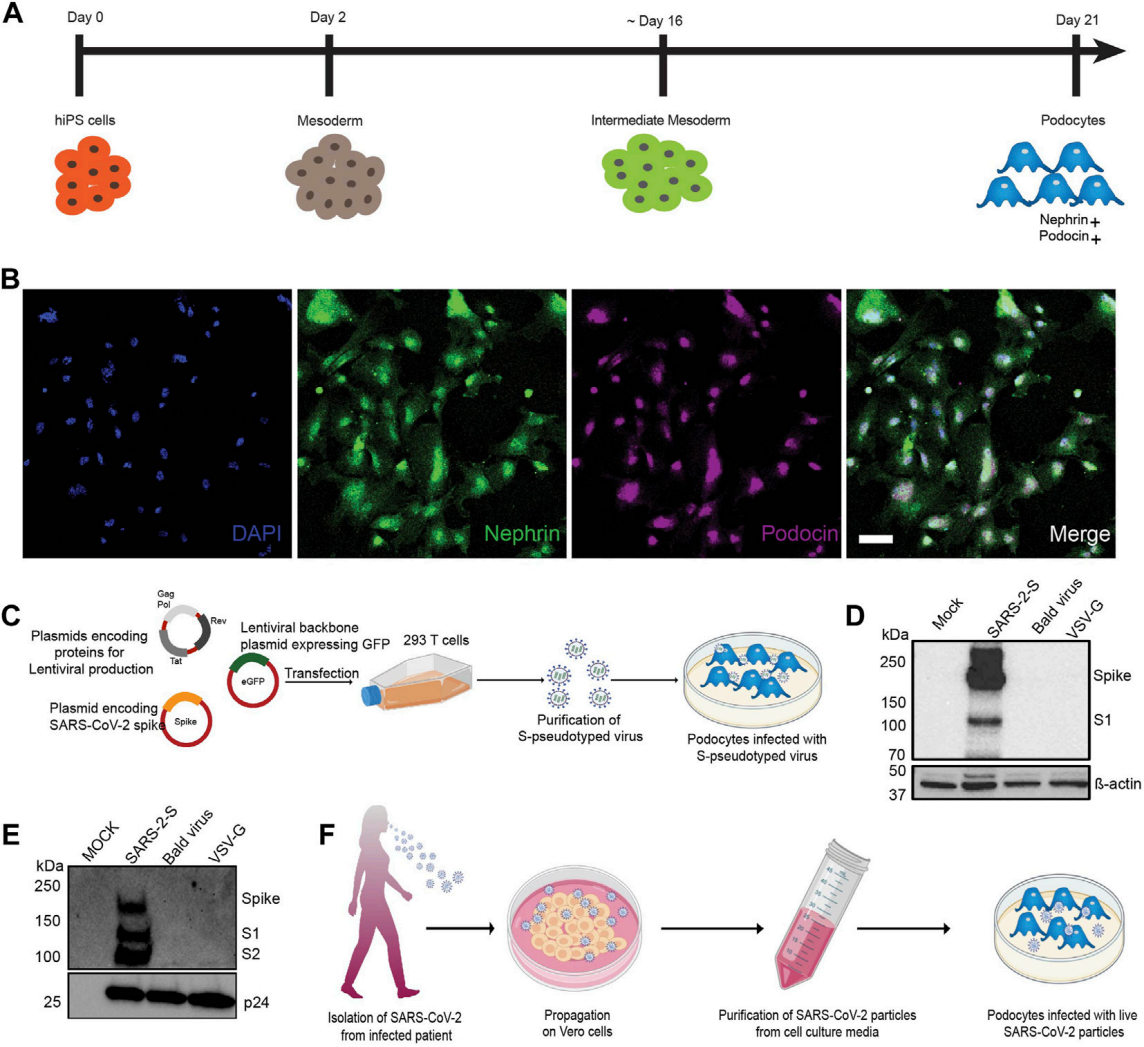
Establishment of a method to examine susceptibility of human iPS cell-derived podocytes to SARS-CoV-2 infection. **(A)** Schematic overview of the protocol for the differentiation of mature podocytes from human iPS cells; adapted from (Musah et al., 2017). **(B)** Human iPS cell-derived podocytes express the lineage specific markers nephrin (green) and podocin (magenta). Cells were counterstained with DAPI (blue) nuclear marker. Scale bar, 100 μm. **(C)** Schematic depicting the lentiviral vectors used to produce S-pseudotyped virus from 293T cells and infection of human iPS cell-derived podocytes *in vitro*. **(D,E)** Western blot confirming the successful transfection of 293T cells and production of S-pseudotyped virus indicated by the presence of Spike protein (190kDa) and its cleavage products S1 (∼110 kDa) in both **(D)** the cell lysates (β-actin used as loading control) and **(E)** purified viral particle (normalized to HIV viral protein p24) with extra band for S2 (∼100 kDa). Mock for figure D represents lysate from untransfected cells while in E, mock represents pseudoviral particle dissolving media (TBS); SARS-2-S represent lysate from cells transfected with spike expressing plasmid or S-pseudotyped particle; Bald virus represent lysate from cells transfected with no envelope plasmid or the resulting pseudovirus particle without viral envelope protein; VSV-G represent lysate from cells transfected with plasmid expressing VSV-G or pseudotyped particle with VSV-G envelope **(F)** Schematic showing propagation of patient-derived SARS-CoV-2 virus in Vero E6 cells followed by purification and infection of human iPS cell-derived podocytes *in vitro*.

Using our previously described protocol, (Musah et al., 2017; Musah et al., 2018; Burt et al., 2020). we differentiated human iPS cells into mature glomerular podocytes (Figure 1A) that exhibited highly specialized morphological features and expressed podocyte-specific markers including nephrin and podocin (Figure 1B).

The spike surface envelope glycoprotein (S) facilitates binding and entry of coronavirus including SARS-CoV and SARS-CoV-2 into cells (Wrapp et al., 2020) and it exhibits capabilities for receptor binding and membrane fusion (Masters and Perlman, 2013; Millet and Whittaker, 2015). We initially employed S-pseudotyped virus to study viral entry and uptake into podocytes. To generate the S-pseudotyped virus, we used an HIV-1-based S-pseudotyped lentiviral vector as illustrated in Figure 1C. Control pseudotyped viruses were generated using the vesicular stromatitis virus glycoprotein (VSV-G; control envelope) plasmid and a “bald” virus lacking an envelope protein. Western blot analysis of 293T cell lysates confirmed the presence of spike protein in only the cells that were transfected with the spike plasmid and not in the cell lysates obtained from VSV-G or bald virus transfection (Figure 1D). Western blot analysis of the 293T cell supernatant from S-pseudotyped particle produced three major bands at 190, 110, and 100 kDa representing the full-length and cleaved S proteins (S1 and S2, respectively) as well as the HIV gag protein (p24). These results confirmed the incorporation of the S protein in the pseudoviral particles and the successful generation of S-pseudotyped virus with the SARS-CoV-2 spike protein (SARS-2-S) (Figure 1E). As expected, the VSV-G and bald pseudoviruses produced only a band for HIV p24 and no band was detected in the mock (medium control) (Figure 1E). Live virus infection of human iPS cell-derived podocytes was performed using SARS-CoV-2 strain USA-WA1/2020 grown on Vero E6 cells as illustrated in Figure 1F.

**FIGURE 2:**
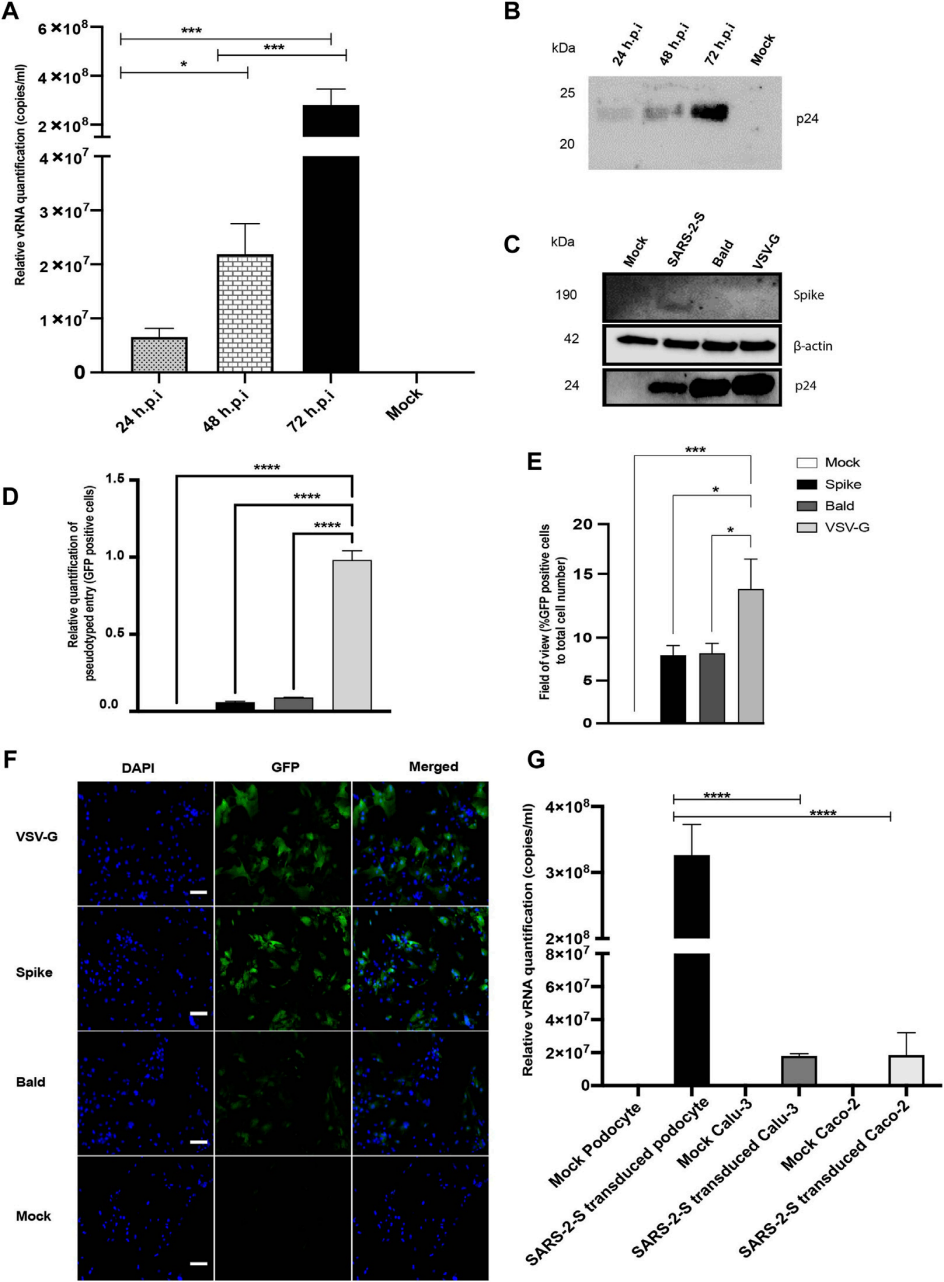
SARS-CoV-2 S-pseudovirus infection of human iPS cell-derived podocytes**. (A)** qRT-PCR analysis using Lenti-X titration kit revealed time-dependent increase in the copies of viral RNA in human iPS cell-derived podocytes infected with the S-pseudovirus. **(B)** Western blot confirmed time-dependent increase of S-pseudovirus particles in human iPS cell-derived podocytes, where p24 (GAG) is a marker for the pseudoviral capsid protein. **(C)** Western blot from cell lysates of mock and infected (S-, Bald and VSV-G pseudotyped) podocytes confirming the presence of SARS-CoV-2 spike proteins in S-pseudotyped infected podocytes, HIV viral protein p24 visible in all pseudotyped infected cells but not in mock and β-actin used as loading control present in all cell lysates **(D)** qRT-PCR data measuring the levels of EGFP using GFP specific primers **(E)** Percentage of EGFP positive cells compared to total cell number **(F)** Immunofluorescent staining showing DAPI, EGFP and merged in pseudotyped infected cells and mock. Scale bar: 100 µm. **(G)** qRT-PCR results showing significantly higher number of S-pseudotyped copies in human iPS cell-derived podocytes compared to Calu-3 and Caco-2 cell lines, 72 h post infection (h.p.i.). The statistical test was done by One-way ANOVA with Sidak’s multiple comparison test. Error bars indicate standard deviation of the mean. Only *p* values of 0.05 or lower were considered statistically significant (*p* > 0.05 [ns, not significant], *p* < 0.05 [*], *p* < 0.01 [**], *p* < 0.001 [***], *p* < 0.0001 [****]).

We initially inoculated human iPS cell-derived podocytes with the S-pseudotyped virus to examine their permissiveness to the virus. The total viral RNA copies in transduced cells were quantified by qRT-PCR using the Lenti-X qRT-PCR titration kit every 24 h post infection (h.p.i) for up to 72 h.p.i. Interestingly, we observed an exponential increase in the number of intracellular RNA copies with each additional day of exposure (Figure 2A), confirming an increase in viral uptake with incubation time. Consistent with these results, Western blot quantification of the relative amount of Gag-p24 taken up by the podocytes each day post-infection (Figure 2B) corroborated the results of relative viral RNA quantification shown in Figure 2A. Furthermore, Western blot analysis of protein lysates generated from uninfected (mock) podocytes, or podocytes infected with S-pseudotyped, bald pseudotyped and VSV-G pseudotyped virus for 72 h revealed bands that correspond to the spike protein only in the lysate from podocytes infected with the S-pseudotyped virus. β-actin (used as loading control) was present in all cell lysates while the band for p24 was observed only in the lysates from the pseudotyped virus infected cells (Figure 2C).

We quantified GFP transcript levels from pseudovirus infected and uninfected cells using qRT-PCR (Figure 2D). When compared to levels of viral uptake (corresponding to the GFP mRNA levels) in VSV-G pseudotyped infected cells, there was a significantly lower uptake in cells infected with S-pseudotyped virus, which is expected since entry of VSV-G typed virus does not require specialized receptors as S-pseudotyped viruses do. GFP-positive cells were imaged by fluorescence microscopy and quantified relative to the total cell counts (Figures 2E,F).

To examine how the levels of viral uptake in the podocytes compare to other organ-specific cell types, we examined pseudoviral uptake in Calu-3 and Caco-2 cells. Intriguingly, there was significantly more viral uptake in the podocytes than Calu-3 and Caco-2 cells (*p*-value < 0.0001 for both) (Figure 2G).

### Live SARS-CoV-2 Virus Infects and Replicates in Human iPS Cell-Derived Podocytes

To explore the susceptibility of human iPS cell-derived podocytes to live SARS-CoV-2, podocytes were incubated with SARS-CoV-2 strain USA-WA1/2020 at MOI of 0.01, 0.1 or 1.0 for 1 h. All steps with live virus were strictly performed in Duke’s BSL3 facility following the guidelines provided by Duke University’s Biosafety committee and the CDC. The range of MOIs chosen was based on previously established models of infection kinetics (Goswami et al., 2021). After 1 h incubation, cells were washed with PBS and then incubated with fresh culture medium for 24, 48 and 72 h (Supplementary Figure S1A). At 24-, 48- and 72-h post-infection, total RNA was extracted from both the cell pellets and the supernatant to evaluate both intracellular and extracellular viral RNA (vRNA) levels.

**FIGURE 3:**
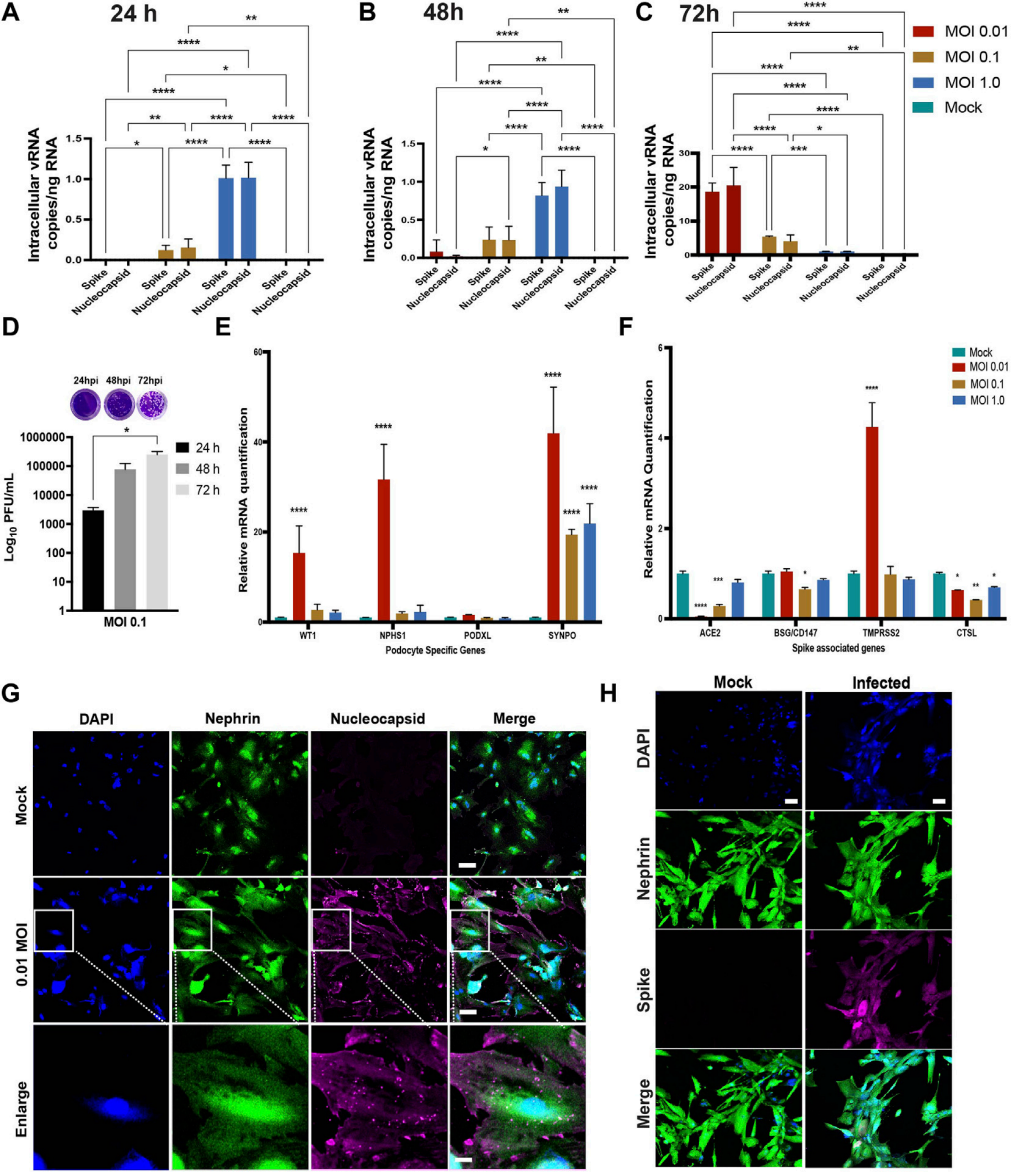
Susceptibility of human iPS cell-derived podocytes to infection by live SARS-CoV-2. qPCR analysis of human iPS cell-derived podocytes infected with SARS-CoV-2 revealed intracellular uptake of the virus for 24 h.p.i **(A)**, 48 h.p.i **(B)** and 72 h.p.i **(C)**. **(D)** plaque assay quantification from supernatant obtained from infected podocytes at 24, 48 and 72 h.p.i. **(E)** qPCR analysis of podocyte-specific genes revealed that both synaptopodin (SYNPO) and podocalyxin (PODXL) are significantly upregulated after infection with SARS-CoV-2 at MOI of 0.01, whereas SYNPO is significantly upregulated at multiple MOIs, and PODXL shows no significant changes with viral infection. **(F)** The expression of spike-associated genes (ACE2, BSG/CD147) and spike processing genes (TMPRSS2, CTSL) are significantly impacted by infection at MOIs of 0.01 and 0.1, respectively. **(G)** Human iPS cell-derived podocytes treated with SARS-CoV-2 (at MOI of 0.01) immunostain positive for Nucleocapsid protein (magenta), indicating successful infection with the virus. The cells were immunostained also for the podocyte marker Nephrin (green) and counterstained with DAPI (blue). Scale bar: 100 µm **(H)** Spike positive cells Nephrin and DAPI as nuclear counterstain in the infected podocytes. Scale bar: 100 µm. One-way analysis of variance (ANOVA) with Sidak’s multiple comparison test was used to determine statistical significance. Only *p* values of 0.05 or lower were considered statistically significant (*p* > 0.05 [ns, not significant], *p* < 0.05 [*], *p* < 0.01 [**], *p* < 0.001 [***], *p* < 0.0001 [****]). Error bars indicate standard deviation of the mean.

We quantified the intracellular and extracellular vRNA copies at 24, 48 and 72 h.p.i. by qRT-PCR using primers specific for SARS-CoV-2 spike and nucleocapsid genes. Analysis of cell pellets collected at 24 and 48 h.p.i demonstrated high levels of viral RNA transcripts in cells infected with MOI of 1.0 (Figures 3A,B). At 72 h.p.i., higher levels of viral RNA transcripts were detected in the cells infected with MOI of 0.01 (Figure 3C). At 72 h.p.i, lower levels of viral RNA were detected in the intracellular fractions from the higher MOI of 0.1 and 1.0 conditions likey due to increased cellular toxicity, leading to a decrease in the number of healthy cells available for additional rounds of viral propagation. These results indicate increased susceptibility of podocytes to primary infection with SARS-CoV-2 even at MOI as low as 0.01. Quantification of the levels of spike and nucleocapsid in cell supernatants revealed an inverse trend (for 72 h.p.i) whereby significantly higher amounts of vRNA was detected in supernatants from cells infected with a MOI of 1.0 than in the cells infected with a MOI of 0.1 or 0.01 at 24, 48 and 72 h.p.i. (Supplementary Figures S1B–D).

We next performed plaque assays to measure the amount of infectious SARS-CoV-2 particles released from infected podocytes (Supplementary Figures S1E–G, respectively). Ten-fold dilutions of each cell supernatant were assessed in duplicates as previously described (Goswami et al., 2021). The number of plaque forming units (PFU) was significantly higher in the cells infected with MOI of 0.1 at 72 h.p.i compared to 24 and 48 h.p.i. (Figure 3D). However, a lower number of PFU was observed in cells infected with MOI of 1.0 compared to cells infected with MOI of 0.1 at 72h.p.i. (Supplementary Figure S1G). These data suggest that the higher vRNA levels observed in Supplementary Figures S1B–D accounts for vRNA released from dying cells that is not incorporated in new infectious particles. Taken together, these results confirm that podocytes are highly permissible host to SARS-CoV-2 infection and replication.

In probing cell viability after SARS-CoV-2 infection, we observed significantly (*p*-value < 0.0001) more cell death in the infected wells compared to controls (Supplementary Figure S2A). We then quantified the mRNA levels of apoptotic genes as well as necroptotic genes to examine whether SARS-CoV-2 can trigger both apoptosis and necroptosis (a form of cell death mediating secretion of inflammatory cytokines) (Pasparakis and Vandenabeele, 2015) in the infected podocytes. It was previously shown that SARS-CoV-2 can trigger apoptosis in Calu-3 cells through caspase-8 activation and that the process was dependent on viral replication (Shufen Li et al., 2020). We observed a significant increase in Caspase 8 mRNA (*p*-value < 0.005) at MOI of 0.01, but not caspase 7, suggesting that the activation of cellular apoptosis is dependent on viral replication (Supplementary Figure S2B). To determine whether SARS-CoV-2 infected podocytes undergo necroptosis, we assessed mRNA expression of the mixed lineage kinase domain-like (MLKL) and the receptor-interacting protein kinase-3 (RIPK3), two effectors of necroptosis. There was a significant upregulation of MLKL (*p*-value < 0.0001) and RIPK3 (*p*-value < 0.0014) in the MOI of 0.01 infected cells (Supplementary Figure S2B), where higher levels of intracellular vRNA were detected (Figure 3C). These results are consistent with a prior report using Calu-3 cells, where activation of necroptosis pathway was shown to be dependent on viral replication (Shufen Li et al., 2020). Conversely, no upregulation of MLKL or RIPK3 mRNA was observed in podocytes infected with either 0.1 or 1.0 MOI of SARS-CoV-2, (Supplementary Figure S2B), presumably due to the lower levels of viral replication in those conditions (Figure 3C). These data suggest that SARS-CoV-2 infection activates necroptosis and apoptosis pathways in podocytes.

### SARS-CoV-2 Infection Alters Podocyte-Specific Gene Expression

Changes in the expression levels of podocyte-specific genes and proteins often correlate with the onset and progression of podocytopathies (Langham et al., 2002; Sung et al., 2006b; Niranjan et al., 2008). Additionally, defects in podocyte structure and function leads to their detachment from the glomerular basement membrane and subsequent loss of the cells into urine, and the onset of glomerulopathies (Kim et al., 2001; Wharram et al., 2005; Matovinović, 2009).

Quantification of podocyte lineage identification genes (WT1, NPHS1, PODXL and SYNPO) after SARS-CoV-2 viral infection at MOI of 0.01 revealed significant increase in WT1, NPHS1 and SYNPO and a moderate increase in PODXL expression levels (Figure 3E). The increased expression of NPHS1 may result from compensatory mechanism to help maintain podocyte physiology post-infection and minimize destabilization of their cellular phenotype as previously reported in a diabetic model of podocyte injury (Sung et al., 2006a). The increase in nephrin gene expression also correlates to the presence of more foot-like processes in the podocytes infected with SARS-CoV-2 at an MOI of 0.01 (Supplementary Figure S2C). However, at MOI of 1.0, we observed changes reminiscent of foot process retraction with a concomitant reduction in nephrin mRNA expression (Figure 3E; Supplementary Figure S2C) indicating a possible maladaptive response with increased viral infection burden. These results indicate that SARS-CoV-2 infection of human iPS cell-derived podocytes leads to dynamic changes in the expression of podocyte-specific genes. Together, our results suggest that infection of podocytes by SARS-CoV-2 results in disrupted molecular profile as well as structural changes which can lead to cell detachment and death (Supplementary Figure S2A).

We also quantified the relative expression of BSG/CD147, ACE2 (given its involvement in SARS-CoV-2 binding and infection of many cell types) (Hoffmann et al., 2020; Shang et al., 2020a; Yanwei Li et al., 2020; Shang et al., 2020b), as well as cell surface protease TMPRSS2 (Wysocki et al., 2021) and endosomal Cathepsin L (CTSL) in podocytes infected with SARS-CoV-2 for 72 h using different MOIs (Figure 3F). We observed that infection at MOI of 0.01 and 0.1 lead to significant reduction in ACE2 expression when compared to uninfected podocytes. Compared to the mock condition, the expression levels of BSG/CD147 remained unchanged for MOI of 0.01 and 1.0, but decreased significantly when the podocytes were infected at an MOI of 0.1. Additionally, TMPRSS2 expression was significantly increased with SARS-CoV-2 infection at MOI of 0.01 but remained relatively similar to the mock condition when the cells were infected at MOI of 0.1 and 1.0. CTSL expression was significantly reduced in all three MOIs. It has been previously shown that COVID-19 infection associates with decreased ACE2 expression due to the internalization of the virus-receptor complex (Gheblawi et al., 2020). Our results show that at MOI of 0.01, expression of ACE2 decreases but that of TMPRSS2 increases, suggesting enhanced enzymatic activity necessary to cleave Spike for processing. These results also show that the low MOI of 0.01 is sufficient for the infection of human iPS cell-derived podocytes with SARS-CoV-2 and indicate that SARS-CoV-2 infection of the podocytes leads to dynamic changes in the expression of spike-binding factors (Figure 3F) as well as podocyte-specific genes (Figure 3E).

Immunofluorescence analysis of the SARS-CoV-2 infected human iPS cell-derived podocytes showed positive immunostaining of the nucleocapsid and spike proteins, suggesting the presence of viral proteins within the cytoplasm even at low MOI of 0.01 (Figures 3G,H). This result further confirmed our observation that SARS-CoV-2 can establish active infection in human iPS cell-derived podocytes. The infected podocytes also exhibited plaque-like regions (Supplementary Figure S2D) and pronounced DAPI staining and spreading indicating more nuclear content in SARS-CoV-2 infected podocytes compared to the mock samples (Figures 3G,H; Supplementary Figure S2E). Changes in nuclear content of the podocytes correlates with enhanced viral replication (De Wilde et al., 2017), that could have led to the genotypic changes in these cells. We used the JACoP plug-in for ImageJ to set thresholds for colocalization analysis and to derive the Pearson’s correlation coefficient (Bolte and Cordelières, 2006). We calculated the Pearson’s coefficient between ACE2 and BSG/CD147 in the control podocyte and obtained a value of 0.81. This confirms a strong positive correlation between ACE2 and BSG/CD147. We then checked for the coefficient between SARS-CoV-2 protein Nucleocapsid and BSG/CD147 in the infected sample and obtained a value of 0.698 which indicates a positive relationship between the molecular components. The coefficient for SARS-CoV-2 protein Nucleocapsid and ACE2 in the infected sample was found to be 0.581, indicating a less positive correlation, which could be due to the reduced expression of ACE2 in infected samples as previously reported in an independent study showing that ACE2 expression is altered in disease conditions or during viral infections (Kuba et al., 2005; Glowacka et al., 2010; Gheblawi et al., 2020). Representative images from our calculations of the Pearson’s correlation coefficient are shown in Supplementary Figure S3A for ACE2 and BSG/CD147, Supplementary Figure S3B for Nucleocapsid and BSG/CD147 and Supplementary Figure S3C for Nucleocapsid and ACE2.

### Human iPS Cell-Derived Podocytes Express Several Spike-Interacting Factors

**FIGURE 4:**
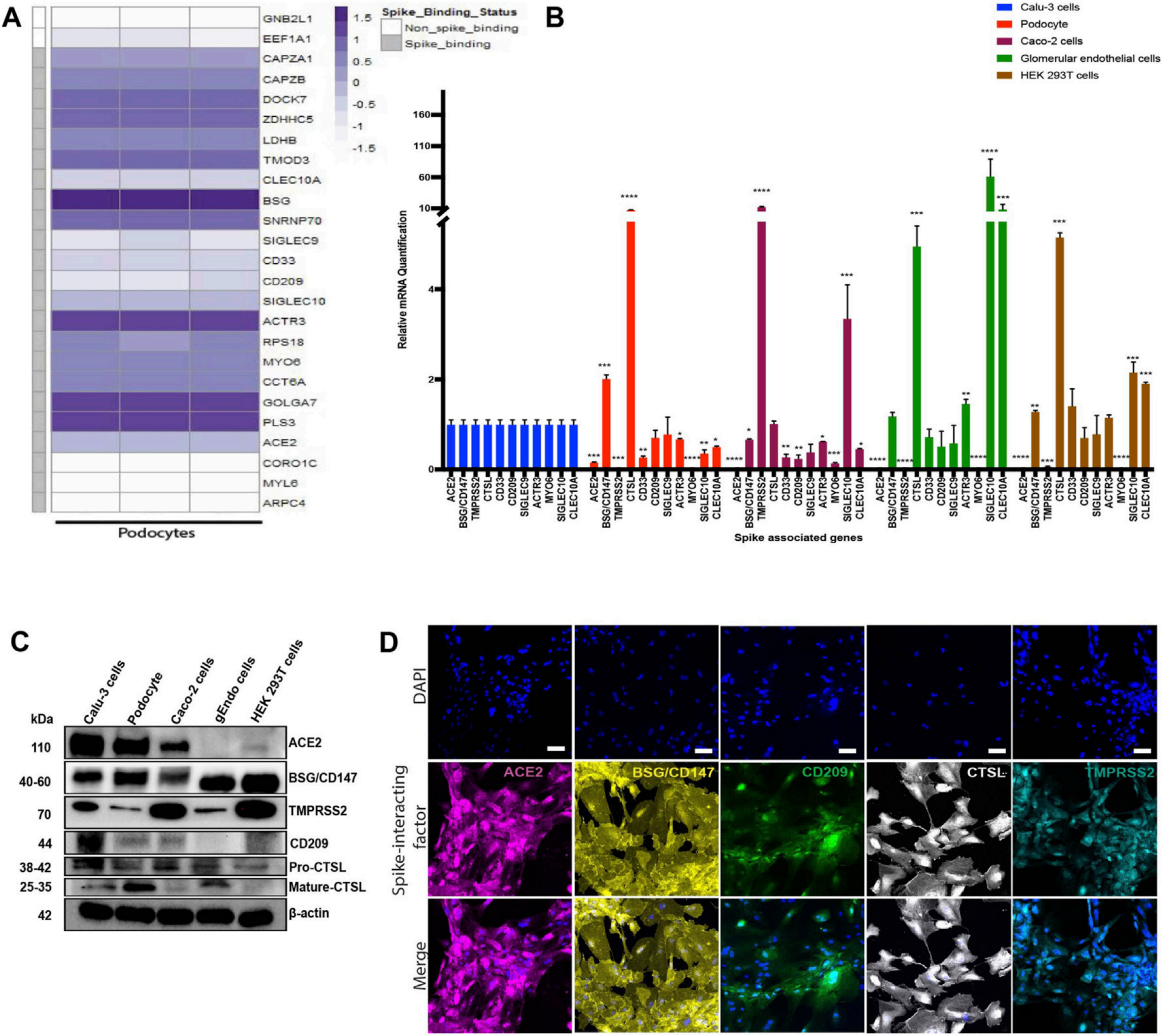
Transcriptomic and protein level analyses of spike-interacting factors in mature podocytes differentiated from human iPS cells. **(A)** Heatmap showing expression levels of spike associated genes from three biological human iPS cell-derived podocyte samples. SIGLEC9, Sialic acid-binding Ig-like lectin 9; CAPZA1, F-actin-capping protein subunit alpha-1; CLEC10A, C-type lectin domain family 10 member A; CD33, Myeloid cell surface antigen CD33; TMOD3, Tropomodulin-3; ACE2, Angiotensin Converting Enzyme 2; BSG/CD147, Basigin/CD147 molecule; CD209, CD209 Antigen; MYO6, Unconventional myosin-VI; PLS3, Plastin-3; LDHB, L-lactate dehydrogenase B chain; GNB2L1/RACK, Receptor of activated protein C kinase 1; SNRNP70, U1 small nuclear ribonucleoprotein 70 kDa; DOCK7, Dedicator of cytokinesis protein 7; RPS18, 40S ribosomal protein S18; CAPZB, F-actin-capping protein subunit beta; GOLGA7, Golgin subfamily A member 7; ZDHHC5, Palmitoyltransferase ZDHHC5; SIGLEC10, Sialic acid-binding Ig-like lectin 10; ACTR3, Actin-related protein 3; MYL6, Myosin light polypeptide 6; CORO1C, Coronin-1C; ARPC4, Actin-related protein 2/3 complex subunit 4; CCT6A, T-complex protein 1 subunit zeta; **(B)** qPCR quantification of nine of the human spike-associated gene from **(A)** including Transmembrane Serine Protease 2 (TMPRSS2) or cathepsin L (CTSL) in human iPS cell-derived podocytes, Calu-3, Caco-2, glomerular endothelial cells, and HEK 293T cells (normalized to Calu-3 groups). **(C)** Western blot analysis to evaluate protein expression of ACE2, BSG/CD147, CD209, TMPRSS2 and CTSL in the different cell type used; Calu-3, human iPS cell-derived podocytes, Caco-2, glomerular endothelial (gEndos) cells, and HEK 293T cells. **(D)** Immunocytochemistry analysis of ACE2, BSG/CD147, CD209, TMPRSS2 and CTSL expression in iPS cell-derived podocytes. Scale bar: 100 µm. One-way analysis of variance (ANOVA) with Sidak’s multiple comparison test was used to determine statistical significance. Only *p* values of 0.05 or lower were considered statistically significant (*p* > 0.05 [ns, not significant], *p* < 0.05 [*], *p* < 0.01 [**], *p* < 0.001 [***], *p* < 0.0001 [****]). Error bars indicate standard deviation of the mean.

Previous studies have identified specific host factors that can facilitate entry of SARS-CoV-2 virus into various tissues and cell types (Cantuti-Castelvetri et al., 2020; Vankadari and Wilce, 2020; Wang et al., 2020; Amraei et al., 2021). To examine whether iPS cell-derived podocytes express host factors that can facilitate entry of SARS-CoV-2 virus, we first explored BioGRID (a database of molecular interactions) and identified twenty-four spike-interacting factors involved in SARS-CoV-2 binding and processing (Supplementary Figure S4A; Supplementary Table S3). We then examined the gene expression levels of the twenty-four spike-interacting factors in human iPS cell-derived podocytes using our previously generated microarray data (Figure 4A) (Musah et al., 2018). Intriguingly, the podocytes expressed twenty (out of twenty-four) spike-interacting factors (Figure 4A; Supplementary Table S3). These results indicate that human iPS cells possess many of the factors involved in SARS-CoV-2 binding and processing, which further supports our data from above showing high SARS-CoV-2 infectivity in the podocytes.

### Comparative Analysis of Spike Interacting Factors in Podocytes and Other Cell Lines

ACE2 is expressed in a variety of human tissues and has been shown to function by counter-balancing the renin-angiotensin-aldosterone system (Hikmet et al., 2020; Yanwei Li et al., 2020). We quantified the basal mRNA expression levels of ACE2, BSG/CD147 and other spike-associated genes in different human cell types (Figure 4B). The expression of these genes is important for the uptake and cleavage of the spike glycoprotein, fusion of SARS-CoV-2 and cell membranes, and subsequent release of viral genome into the cytoplasm of an infected cell (Millet and Whittaker, 2015).

Studies have shown that some cells with little to no ACE2 expression can still be infected with SARS-CoV-2 (Hikmet et al., 2020; Singh et al., 2020), suggesting that other class of receptors might facilitate viral infection in ACE2-deficient cell types. Additionally, it has been shown that the expression levels of viral uptake receptors can vary significantly between different cell types (Cantuti-Castelvetri et al., 2020; Wang et al., 2020). These findings suggest that ACE2 may not be the only receptor for SARS-CoV-2 in some cells, and that there could be multiple mechanisms for viral infection and processing. We examined expression levels of several of the factors in multiple cell types (podocytes, Calu-3, Caco-2, glomerular endothelium, and HEK 293T) to help understand the levels of tissue or cell-type specificity (Figure 4B). We found that there was no ACE2 expression in HEK 293T cells and glomerular endothelial cells and only little expression in Caco-2 cells compared to Calu-3. Intriguingly, ACE2 expression in human iPS cell-derived podocytes is approximately 10 times lower than the expression level in Calu-3 cells and slightly higher than the expression level in Caco-2 cells.

We observed a significantly low expression level of TMPRRS2 in the podocytes compared to Calu-3 cells (Figure 4B). A lower level expression of TMPRRS2 was also previously reported for cardiomyocytes derived from human embryonic stem cell (hESC-CMs) and a different endosomal viral processing protease was shown to be much more highly expressed (Marchiano et al., 2021). Our qPCR results showed a significantly higher expression of CTSL in podocytes when compared to Calu-3 cells (Figure 4B), suggesting that the mechanism of SARS-CoV-2 entry in podocytes might be different from the TMPRRS2-dependent mechanisms observed in lung epithelial cells (Hoffmann et al., 2020; Shang et al., 2020a).

It is likely that SARS-CoV-2 infection of podocytes relies on ACE2, BSG/CD147 and other genes that might direct membrane fusion and/or entry through the endo-lysosomal pathway. Together, these results show that human iPS cell-derived podocytes express proteins that make them susceptible to SARS-CoV-2 infection, like human iPS cell derived cardiomyocytes (Sharma et al., 2020; Marchiano et al., 2021). The expression of CD209, which is recognized as an alternative receptor for lung and kidney epithelial and endothelial cells (Amraei et al., 2021), was comparable between Calu-3 cells, human iPS cell-derived podocytes, glomerular endothelial cells and HEK 293T cells but significantly lower in Caco-2 cells (Figure 4B). The mRNA expression of the other genes, SIGLEC9, ACTR3, MYO6, SIGLEC10 and CLEC10A, varied between the different cell types (Figure 4B). We then validated the relative protein level expression of three uptake (ACE2, BSG/CD147, CD209) and two processing (TMPRSS2, and CTSL) factors in podocytes and other cell types (Calu-3, Caco-2, glomerular endothelia and 293T cells). Western blot analysis confirmed higher expression of ACE2 protein in Calu-3 and then podocytes, and little to no expression in glomerular endothelial cells (gEndos) (Figure 4C). This result also demonstrated that podocytes express more ACE2 than Caco-2 cells, consistent with the gene expression data in Figure 4B. Additionally, the Western blot data for BSG/CD147 corroborated the mRNA data showing higher expression in podocytes than Calu-3 and Caco-2. Figure 4C also shows the expression of CD209 in podocytes and the other cell types. We observed relatively low gene and protein level expression of TMPRSS2 in podocytes when compared to the other cell types. Although mature CTSL protein is present in podocytes, Calu-3 and glomerular endothelia cells, pro-CTSL is present in all the cell types which may explain the presence of the CTSL mRNA in all the cell types even when they do not express mature CTSL protein. It has been suggested that SARS-CoV-2 entry into host cells depends on the presence of cholesterol-rich lipid rafts, which facilitates membrane fusion through proteases such as TMPRSS2 or endosomal pathway using Cathepsin B&L (Palacios-Rápalo et al., 2021). Our results suggests low levels of TMPRSS2 expression in podocyte, and we speculate that the use of BSG/CD147 as a receptor and CTSL as a processing enzyme for viral entry mediated by S protein might be a preferred mechanism for the podocytes. Finally, expression of all these proteins was also validated using immunocytochemistry in podocytes (Figure 4D), Calu-3 (Supplementary Figure S4B), Caco-2 (Supplementary Figure S4C), glomerular endothelial (Supplementary Figure S4D) and 293T (Supplementary Figure S4E) cells. These findings strongly indicate that human kidney podocytes employ multiple spike-binding receptors (in addition to ACE2) for SARS-CoV-2 viral uptake.

### Receptor Antibodies can Reduce SARS-CoV-2 Pseudovirus Entry Into Human iPS Cell-Derived Podocytes

Based on the hypothesis that SARS-CoV-2 could exploit both ACE2 and BSG/CD147 receptors for viral uptake in human iPS cell-derived podocytes, we investigated whether antibodies against these two spike receptors can block the entry of S-pseudotyped virus in the cells. We used anti-hACE2 and anti-CD147 (anti-BSG) antibodies at varying concentrations to block ACE2 and BSG/CD147 receptors from interacting with pseudoviral particles.

**FIGURE 5:**
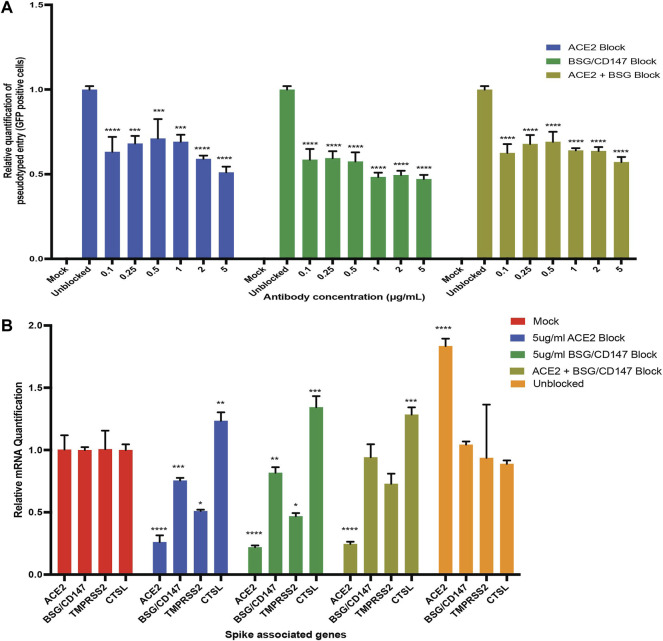
Antibody blocking reveal roles of ACE2 and BSG/CD147 receptors in viral uptake. **(A)** qPCR quantification of S-pseudotyped entry relative to antibody concentration (normalized to unblocked samples). Human iPS cell-derived podocytes were incubated with different dilutions of anti-ACE2, anti-BSG or both for an hour followed by infection with S-pseudotyped virus at MOI 0.02 for 60 h **(B)** qPCR quantification of Spike binding receptor genes (ACE2, BSG/CD147) and Spike processing factor genes (TMPRSS2, CTSL) at 5 µg/ml for both anti-ACE2 antibody blockage and anti-BSG antibody blockage showing significant changes in gene expression with optimal receptor blockage when compared to unblocked samples (normalized to mock groups). One-way analysis of variance (ANOVA) with Sidak’s multiple comparison test was used to determine statistical significance. Only *p* values of 0.05 or lower were considered statistically significant (*p* > 0.05 [ns, not significant], *p* < 0.05 [*], *p* < 0.01 [**], *p* < 0.001 [***], *p* < 0.0001 [****]). Error bars indicate standard deviation of the mean.

When the podocytes were blocked with ACE2 or BSG/CD147 antibody at varying concentrations (from 0.1 to 5 µg/ml), we observed a concentration-dependent and statistically significant decrease in viral uptake (Figure 5A). The highest concentration of the antibody (5 µg/ml) was most effective for blocking the receptors while significantly (*p*-value < 0.0001) reducing cellular uptake of the virus (Figure 5A). These results confirm that ACE2 and BSG/CD147 facilitate S-pseudotyped viral update in podocytes. The observed high expression of BSG/CD147 receptor in podocytes revealed by microarray (Figure 4A), qPCR data (Figure 4B), Western blotting (Figure 4C) and immunocytochemistry (Figure 4D) further suggest that these receptors interact with the spike protein of SARS-CoV-2 and facilitate its uptake and entry into the cells. As a result, blocking with anti-BSG/CD147 significantly decreased viral uptake similar to that observed with ACE2 blocking.

To examine if blocking with both anti-ACE2 and anti-BSG had a synergistic effect, the podocytes were simultaneously pretreated with both the antibodies and then infected with the virus. We observed similar trend where the lowest viral uptake was at 5 µg/ml (Figure 5A). This result suggests that both ACE2 and BSG/CD147 are involved in SARS-CoV-2 internalization in human iPS cell-derived podocytes.

Next, we examined whether the expression of the key viral entry genes, ACE2, BSG/CD147, TMPRSS2 and CTSL are altered during antibody blocking. Exposure of podocytes to the pseudoviral particles after blocking with 5 µg/ml anti-ACE2 antibodies showed significant downregulation of BSG/CD147 and TMPRSS2 transcripts, but upregulation of CTSL compared to unblocked cells (Figure 5B). Similarly, exposure of podocytes to the pseudoviral particles after blocking with 5 µg/ml anti-CD147 antibodies showed significant downregulation of ACE2 and TMPRSS2 transcripts, but upregulation of CTSL compared to the unblocked cells. The successful blocking of ACE2 or CD147 anti-ACE2 or anti-CD147 (anti-BSG) antibodies respectively was as expected and is also shown in Figure 5B. However, when podocytes were exposed to 5 µg/ml of both anti-ACE2 and anti-CD147 (anti-BSG) antibodies, the level of ACE2 decreased as expected, but the level of BSG/CD147 unexpectedly remained high and was comparable to the unblocked cells (Figure 5B). The level of CTSL was higher than the unblocked cells when they were simultaneousy exposed to both antibodies.

These findings supports recent report that abundance of ACE2 correlates with with the levels of BSG/CD147 and that the two receptors are being co-regulated (Fenizia et al., 2021). Since viral entry was not fully inhibited when both receptors were blocked, it is possible that other types of receptors facilitate SARS-COV-2 viral entry in podocytes when ACE2 and BSG/CD147 are blocked.

## Discussion

The global pandemic caused by SARS-CoV-2 has resulted in the loss of millions of lives and caused devastating social and economic burdens. The disease mostly presents as a respiratory illness, similar to viral pneumonia, and in more severe cases as acute respiratory distress syndrome (ARDS) (De Wilde et al., 2017; Gupta et al., 2020; Simoneau and Ott, 2020). In addition, several COVID-19 patients simultaneously experience renal, cardiac, neurological, digestive, and/or pancreatic complications (Gupta et al., 2020; Singh et al., 2020). Although the onset of acute kidney injury and collapsing glomerulopathy (Velez et al., 2020) have been clinically associated with the severe form of COVID-19 (Braun et al., 2020), it remains unknown how the kidneys are specifically targeted by the virus, and whether podocytes—the specialized epithelial cells that help form the blood filtration barrier in the kidneys—can be directly infected by the virus.

To address some of these important questions, we have shown in this paper that human iPS cell-derived podocytes are highly susceptible to SARS-CoV-2 infection. Initial viral infection of cells at low MOIs (<1.0) allows additional rounds of virus replication in in a 2D culture, as the replicating virus can enter adjacent cells that were not infected during primary infection. Indeed, we observed higher levels of intracellular vRNA and higher number of PFU at 72 h.p.i. in cells infected with MOI of 0.01 of SARS-CoV-2 compared to MOI of 1.0. These results are relevant and consistent with a recent report showing that SARS-CoV-2 isolated from COVID-19 autopsied kidney could extensively infect kidney tubular cells *in vitro* and lead to extensive viral replication that produced 1000-fold increase in the amount of viral RNA, confirming the presence of infectious virus in the kidneys (Braun et al., 2020).

Due to the lower ACE2 mRNA expression in podocytes when compared to Calu-3, we evaluated the extent of viral infections using various MOIs from 0.01 to 1.0, as it was initially unknown if human iPS cell-derived podocytes would be permissive to direct infection with SARS-CoV-2. Typically, high MOIs (e.g., 1.0 or more) are required if the cell type is minimally permissive to viral infection (Simoneau and Ott, 2020) as observed for some organoid models (Huang et al., 2020; Monteil et al., 2020) within which cell-type-specific responses could not be fully evaluated due to high levels of heterogeneity (Bar-Ephraim et al., 2020). We show, however, that specialized podocytes derived from human iPS cells can be directly infected with SARS-CoV-2 at low MOIs of 0.01–1.0 (Figures 3A–C). The level of the cellular uptake of viral particles can be quantified using multiple assays including immunofluorescence microscopy (for the structural protein N or S, or against dsRNA intermediate), by quantitative RT-PCR of vRNA and plaque assay to quantify infection in the supernatant (Simoneau and Ott, 2020). Indeed, we confirmed viral uptake by the human iPS cell-derived podocytes using qRT-PCR at 24, 48 and 72 h.p.i. (Figures 3A–C), plaque assay (Figure 3D) and immunofluorescence microscopy analysis with both anti-N and -S antibodies (Figures 3F,G). In addition, our investigation of how infection alters the expression of the spike-associated genes revealed a significant reduction in ACE2 expression when compared to uninfected samples (Figure 4B). This is in line with down regulation of ACE2 expression upon SARS-CoV spike protein binding which promotes lung injury (Glowacka et al., 2010) as well as reduction in ACE2 expression due to SARS-CoV replication in Vero cells (Kuba et al., 2005).

Our results also revealed that human iPS cell-derived podocytes express lower levels of ACE2 (Mizuiri and Ohashi, 2015) and TMPRSS2 when compared to Calu-3 (Figures 4B,C). Since SARS-CoV-2 -host interaction is vital for viral pathogenesis, ultimately determining the outcome of infection (De Wilde et al., 2017), and the functional activity of the virus depends on the proteolytic processing during cell entry (Simmons et al., 2005; Kang et al., 2020), we next sought to identify other factors that could mediate viral entry in iPS cell-derived podocytes. We utilized BioGRID analysis to gain insight into SARS-CoV-2—host interactions by mapping out spike-binding proteins expressed in podocytes. Viral processing factors have been shown to be co-expressed with the type of spike binding receptor used by a given cell (Muus et al., 2020; Marchiano et al., 2021; Wysocki et al., 2021). In this study, we identified BSG/CD147 as a mediator of SARS-CoV-2 entry into podocytes along with ACE2 (Figure 5A)**.** Our results add to the repertoire of cells that employ BSG/CD147 as a receptor for viral entry as recently reported for Calu-3 cells (Fenizia et al., 2021). Thus, our results indicate that SARS-CoV-2 employs multiple receptors and viral processing mechanisms to directly infect human iPS cell-derived podocytes.

The importance of employing cell models with mature phenotypes, which has historically been difficult for organoids and other iPS derived cell models, cannot be over-emphasized. For example iPS cell-derived renal organoids generate glomeruli with transcriptomic signatures similar to fetal stages (Homan et al., 2019) which poses a question as to whether human iPS cell-derived cells can recapitulate the biology of SARS-CoV-2 infection in adults since vertical infection of the fetus is still unclear (Lamouroux et al., 2020) but remains a possibility (Chen et al., 2020; Muus et al., 2020). Furthermore, this points to the tissue-specific viral tropisms that may determine whether a productive infection is established in any given tissue. Therefore, it is important to understand these non-canonical SARS-CoV-2 entry-mediating proteins (i.e., other than ACE2 and TMPRSS2) so that we can establish effective methods to block viral replication in those tissues in which ACE2/TMPRSS are poorly expressed or not employed for viral infection.

Aside from being a receptor for SARS-CoV-2, ACE2 plays important role in different tissues in controlling blood pressure (Wysocki et al., 2010; Wang et al., 2020; Wysocki et al., 2021) or preventing heart failure and kidney injury (Wong et al., 2007; Batlle et al., 2012; Mizuiri and Ohashi, 2015). As such, development of drugs to block ACE2 might have a negative effect on its other protective functions. BSG/CD147 has been implicated in tumor metastasis, inflammation and viral infection (Pushkarsky et al., 2001; Chen et al., 2005; Douglas et al., 2014; Zhang et al., 2018) and also previously shown to facilitate SARS-CoV invasion in host cells (Chen et al., 2005; Wang et al., 2020). Our results shows that antibody blocking of BSG/CD147 receptors significantly reduces SARS-CoV-2 viral uptake in human iPS cell-derived podocytes. Thus, BSG/CD147 could potentially be a useful target for antiviral therapeutics including those aimed to address SARS-CoV-2 infections and COVID-19 disease. On the other hand, BSG/CD147, which belongs to the Ig superfamily is expressed in several tissues like the brain, heart, liver, kidney etc and might play a complex role in COVID-19 and possibly contribute to the worse prognosis of patients with other co-morbidities (Radzikowska et al., 2020; Fenizia et al., 2021). Given the high coregulation between ACE2 and BSG/CD147, it might be beneficial to explore additional cell-entry mechanisms to inform future therapeutic strategies for the prevention and treatment of SARS-CoV-2 infection in human tissues and organs.

## Data Availability

The original contributions presented in the study are included in the article/Supplementary Material, further inquiries can be directed to the corresponding author.
