# Supplementary material for: SARS-CoV-2 Employ BSG/CD147 and ACE2 Receptors to Directly Infect Human Induced Pluripotent Stem Cell-Derived Kidney Podocytes
Source: Front Cell Dev Biol. 2022 Apr 20;10:855340. doi: 10.3389/fcell.2022.855340 (PMC9065256; doi:10.3389/fcell.2022.855340)
Supplement: Supplementary file 1 [file Table2.DOCX]

**Supplementary Table 2**

**Ordering information and working conditions of used antibodies for western blot**

| Antibody | Company | Catalog | Dilution | Conditions |
| --- | --- | --- | --- | --- |
| Spike | ProSci | 3525 | 1:1000 | Overnight, 4 °C |
| ACE2 | R&D systems | AF933 | 1µg/ml | Overnight, 4 °C |
| BSG/CD147 | R&D systems | MAB3195 | 1µg/ml | Overnight, 4 °C |
| TMPRSS2 | Santa Cruz Biotechnology | sc-515727 | 1:500 | Overnight, 4 °C |
| DC-SIGN/CD209 | Santa Cruz Biotechnology | sc-65740 | 1:1000 | Overnight, 4 °C |
| Cathepsin L | Santa Cruz Biotechnology | sc-32320 | 1:1000 | Overnight, 4 °C |
| β-actin | Cell Signaling Technologies | 4967S | 1:1000 | Overnight, 4 °C |
| P24 | Santa Cruz Biotechnology, | sc-69728 | 1:500 | Overnight, 4 °C |
| Anti-mouse HRP | Cell Signaling Technologies | 7076 | 1:5000 | 1 h, r.t. |
| Anti-rabbit HRP | Cell Signaling Technologies | 7074 | 1:5000 | 1 h, r.t. |
| Anti-goat HRP | R&D Biosystems | HAF017 | 1:1000 | 1 h, r.t. |
