# Supplementary material for: SARS-CoV-2 Employ BSG/CD147 and ACE2 Receptors to Directly Infect Human Induced Pluripotent Stem Cell-Derived Kidney Podocytes
Source: Front Cell Dev Biol. 2022 Apr 20;10:855340. doi: 10.3389/fcell.2022.855340 (PMC9065256; doi:10.3389/fcell.2022.855340)
Supplement: Supplementary file 2 [file Table3.DOCX]

**Supplementary Table 3**

**SARS-CoV-2 interacting process expressed in podocytes (from BioGRID and podocyte microarray data).**

Below are the list of genes whose proteins are SARS-CoV-2 spike-interacting as identified from the BioGRID experimental database (Oughtred et al., 2019). Background-subtracted and normalized (Lim et al., 2007) expression levels (microarray fluorescence intensity) in human iPS cell-derived podocytes are indicated in the rightmost column.

| **Gene** | **Full name** | **Average expression (RMA-normalized fluorescence) in podocytes (microarray)** |
| --- | --- | --- |
| SIGLEC9 | **Sialic acid-binding Ig-like lectin 9** | 3.057 |
| CAPZA1 | **F-actin-capping protein subunit alpha-1** | 6.191 |
| CLEC10A | **C-type lectin domain family 10 member A** | 3.818 |
| CD33 | **Myeloid cell surface antigen CD33** | 4.046 |
| TMOD3 | **Tropomodulin-3** | 8.085 |
| ACE2 | Angiotensin Converting Enzyme 2 | 4.851 |
| BSG/CD147 | Basigin/CD147 molecule | 10.991 |
| CD209 | CD209 Antigen | 3.276 |
| MYO6 | **Unconventional myosin-VI** | 7.610 |
| PLS3 | **Plastin-3** | 9.309 |
| LDHB | **L-lactate dehydrogenase B chain** | 6.956 |
| GNB2L1 | **Receptor of activated protein C kinase 1** | Not detected (0) |
| SNRNP70 | **U1 small nuclear ribonucleoprotein 70 kDa** | 8.005 |
| DOCK7 | **Dedicator of cytokinesis protein 7** | 8.279 |
| RPS18 | **40S ribosomal protein S18** | 6.804 |
| CAPZB | **F-actin-capping protein subunit beta** | 7.016 |
| GOLGA7 | **Golgin subfamily A member 7** | 9.379 |
| ZDHHC5 | **Palmitoyltransferase ZDHHC5** | 8.468 |
| SIGLEC10 | **Sialic acid-binding Ig-like lectin 10** | 4.770 |
| ACTR3 | **Actin-related protein 3** | 10.071 |
| MYL6 | **Myosin light polypeptide 6** | Not detected (0) |
| CORO1C | **Coronin-1C** | Not detected (0) |
| ARPC4 | **Actin-related protein 2/3 complex subunit 4** | Not detected (0) |
| CCT6A | **T-complex protein 1 subunit zeta** | 6.9179 |

RMA= Robust Microarray Average

**Reference**

Lim, W.K., Wang, K., Lefebvre, C., and Califano, A. (2007). Comparative analysis of microarray normalization procedures: effects on reverse engineering gene networks. *Bioinformatics* 23(13)**,** i282-i288.

Oughtred, R., Stark, C., Breitkreutz, B.-J., Rust, J., Boucher, L., Chang, C., et al. (2019). The BioGRID interaction database: 2019 update. *Nucleic Acids Research* 47(D1)**,** D529-D541. doi: 10.1093/nar/gky1079.
